# Risks for Mental Illness in Indigenous Australian Children: A Descriptive Study Demonstrating High Levels of Vulnerability

**DOI:** 10.1111/1468-0009.12263

**Published:** 2017-06-06

**Authors:** ASTERIE TWIZEYEMARIYA, SOPHIE GUY, GARETH FURBER, LEONIE SEGAL

**Affiliations:** ^1^ School of Health Sciences University of South Australia

**Keywords:** mental health, indigenous children, multiple disadvantage, Australia

## Abstract

**Policy Points:**

- The developmental origins understanding of mental illness suggests the possibility of prevention, through addressing childhood adversities.
- More than 43% of Indigenous Australian children aged 6 to 10 years have 6 or more risk factors for mental illness in adulthood, and 23% are experiencing current psychological distress.
- Substantial risk is already present in infancy (eg, 67% exposed to 3 or more stressful family life events, and 42.5% are not living with both birth parents).
- An integrated service system response that can both offer high‐level therapeutic services and address associated multiple adversities, from conception to late adolescence, is urgently needed to address current psychological distress in Indigenous Australian children and to reduce the future burden of mental illness.

**Context:**

Mental illness is a high source of disease burden across the globe. Mental illness is now understood as largely developmental, with its genesis at least in part in adverse childhood experiences. We sought to estimate the prevalence of childhood risks for poor mental health in Indigenous Australian children, noting that Indigenous Australians by virtue of their history of traumatic colonization and dispossession, child removal, and racism are potentially at greater risk.

**Methods:**

We conducted a descriptive study of the modifiable risks and adversities associated with mental illness in Australian Indigenous children (infancy to 10 years), using data from the Longitudinal Study of Indigenous Children (LSIC), a cohort of 1,671 infants enrolled in 2008 and followed up annually. Risk factors of interest were identified from a comprehensive literature review of childhood determinants of adult mental illness by Fryers and Brugha (2013). The age‐category prevalence of individual risks and a multiple‐risk score were computed using data extracted from 6 waves of LSIC.

**Findings:**

The analytic data comprised 8,378 person‐observations from the first 6 waves. Children in LSIC experience high rates of adversities. In utero, nearly 50% were exposed to smoking and 22% to alcohol. As infants, 42.5% were not living with both birth parents. Over two‐thirds of survey children were in households that had experienced, in the previous 12 months, 3 or more major life events; 22% to 26% lived in households with a drug or alcohol problem; and 18% were exposed to domestic violence (41% were ever‐exposed to age 10). At school, nearly 40% of children were bullied. Over 45% of children aged 6 to 10 years were exposed to 6 or more risks for mental illness. Few children (< 2%) were exposed to low parental warmth and, despite the high exposure to adversities, less than 5% of children report low self‐confidence. This suggests considerable resilience. More than 1 in 4 children had conduct problems in the clinical range and more than 1 in 5 were experiencing high psychological distress.

**Conclusions:**

Indigenous children in Australia face extreme levels of multiple disadvantage, exposing many to current psychological distress and high risk of developing mental illness, despite considerable resilience. For policymakers, this is a call for preventive action targeting the multiple risk factors already present in childhood. An integrated service system offering culturally appropriate, high‐quality early childhood education services, linked to infant, child, adolescent, and family mental health services, and intensive family support services will be crucial in addressing this public health crisis.

Mental health and well‐being is shaped by a broad range of factors, including genetics, family and peer relationships, psychological and physiological functioning, lifestyle, occupation/education, physical environment, socioeconomic status, cultural factors, and the historical and political context.^1^ While the interplay of these factors (positive and negative) can be complex, it is well established that the accumulation of risks and adversities over childhood and adolescence increases the risk of poor mental health and developing a mental illness.^2^, ^3^, ^4^, ^5^, ^6^ Exposure to major life stresses, such as extreme poverty, family violence, child abuse and neglect, and homelessness, in the early stages of life can be particularly harmful to the developing brain^7^, ^8^ and to psychological health in childhood with negative consequences for mental health and well‐being across the lifespan and intergenerationally.^9^ Many of the most potent risk factors cluster in early life, including poor parental mental health and alcohol intake during pregnancy, which can result in developmental delay and poor physical and neurological outcomes.^10^, ^11^, ^12^

Across the globe, the health of indigenous peoples is considerably poorer than that of nonindigenous people. Aboriginal Australians are one of the oldest living cultures in the world^13^ and constitute 3% of the Australian population.^14^ As a lasting legacy of the trauma of colonization, systemic discrimination and racism, separation of families, removal from country, and loss of language and culture, Indigenous Australians show poorer outcomes on a range of measures. These include a 10‐plus‐year difference in life expectancy and higher rates of infant mortality, hospitalizations for chronic conditions, injury, and disability.^15^, ^16^, ^17^, ^18^ The story is similar for mental health. Indigenous Australian adults are 3 times more likely to experience high or very high levels of psychological distress than nonindigenous adults, and hospitalization rates for mental and behavioral disorders are nearly twice the rate for nonindigenous Australians (21.7/1,000 vs 12.9/1,000).^19^, ^20^

Evidence of high rates of psychological distress in aboriginal children is also emerging. The Western Australian Aboriginal Child Health Survey (WAACHS) found that 24% of Western Australian Aboriginal children aged 4 to 17 years showed signs of serious emotional or behavioral difficulties, compared with 14% for the general population.^21^, ^22^ Deaths due to self‐harm among young Indigenous Australians aged 5 to 17 years was over 5 times the rate for nonindigenous young people between 2010 and 2014 (9.3/100,000 vs 1.7/100,000).^23^ Modifiable risks that may contribute to these poor outcomes for young indigenous people include developmental vulnerability, poorer school attainment/attendance, stressful life events (eg, death in the family, serious illness, family breakdown, financial problems, and incarceration), poorer nutrition, higher rates of smoking, alcohol abuse, and housing instability.^22^, ^24^, ^25^, ^26^ In 2014‐2015 an estimated 10% of Aboriginal mothers of infants drank alcohol during pregnancy (a rate half that of 8 years earlier), and just under 30% of Aboriginal and Torres Strait Islanders had experienced homelessness.^25^ It was also the case that over one‐third of Aboriginal and Torres Strait Islander children spoke an indigenous language and nearly 30% spent time with an Aboriginal elder or leader each week.^27^

Detailed analyses of the level of risk exposure for Indigenous Australians especially during the critical early developmental period of infancy are lacking. Furthermore, as risks multiply, the likelihood of poor outcomes increases exponentially.^28^ Describing the prevalence of multiple risks in Indigenous children is crucial for planning services that can respond to complexity in Indigenous families. Such descriptions of multiple risks are not yet available. Understanding the type and level of risk factor exposure in Indigenous Australian children will help guide policy that, for example, strengthens the mental health system for infants and children with the hope of preventing the compounding and escalating risk cycles that lead to entrenched adult mental illness.

The aim of this study is to advance the prevention agenda by describing the prevalence of childhood antecedents of mental illness in Indigenous Australian children, including their exposure to multiple risks. It forms part of a larger research program to apply an evidence‐informed needs‐based planning framework to determine the optimal workforce and service structure required to deliver best practice in preventive mental health care^29^ (complementing recent work by Guy et al^30^ on rates of risk exposure in predominantly nonindigenous Australian children).

## Methods

### Selecting Critical Childhood Risk Factors for Adult Mental Illness

The literature on childhood risk factors for adult mental illness is large and challenging to summarize. This paper adopts the method developed in our previous study on Australian children^30^ that draws on a recent systematic review and summary of the literature by Fryers and Brugha.^10^ They proposed a taxonomy of risk factors for adult mental illness grouped into 10 domains, based on their review of 450 longitudinal studies that explored risk relationships between genetics, environmental factors, individual attributes, and mental health.

We created 8 domains (excluding genetics and combining “psychological disturbances” and “disturbed behaviors”) to guide the selection of variables for designation as childhood risk factors for mental illness:

1. *Neurological vulnerability in childhood*—eg, brain damage, birth complications, intellectual disability, developmental delay
2. *Psychological disturbances and disturbed behaviors in childhood and adolescence*—eg, hyperactivity, conduct problems
3. *Features of personality*—eg, neuroticism or low self‐esteem
4. *Poor school performance—*poor educational achievement
5. *Childhood/family adversity*—life events, eg, physical illness, extreme poverty, parental incarceration
6. *Child maltreatment*—eg, physical abuse, sexual abuse, neglect, witnessing domestic violence
7. *Parenting, parent‐child relationships, and parent psychological distress—*eg, harsh discipline, parental distress
8. *Disrupted families*—eg, divorce and separation, parental substance misuse, family conflict

While the domains were identified from predominantly (although not exclusively) nonindigenous populations from across the globe, categories of risk for poor mental health identified in studies of indigenous populations are similar.^22^ To translate these domains into data items for which prevalence could be established, we matched risk factors covered under these 8 domains against pertinent data items from the Longitudinal Study of Indigenous Children (LSIC).

### The Longitudinal Study of Indigenous Children

The LSIC is an Australia‐wide multistage clustered survey^31^ that aims to examine a broad range of policy‐relevant questions about Indigenous children's development and well‐being. The survey design and implementation involved extensive consultation with Indigenous Australians. As described by the LSIC team, “The Steering Committee has been chaired by an Indigenous leader, Professor Mick Dodson AM, since 2003, and includes a majority of Indigenous members. Consultation and negotiation have been integral to the research methodology in ensuring the genuine participation of Indigenous people and a sense of local ownership. LSIC held extensive consultations with Indigenous communities in urban, regional, and remote areas to inform the development of the study design and content.”^32^ The research questions guiding the LSIC study were thus framed within Indigenous contexts, and the data items and survey instrument items were selected for their relevance to Indigenous Australians.

Development of the LSIC sample involved a 2‐stage approach. In the first stage, 11 sites across Australia, ranging from very remote communities to capital cities, were purposively selected to be representative of the socioeconomic and community environments where Aboriginal and Torres Strait Islander children live. The second stage involved the recruitment of Indigenous children (and their families) in those areas.

The majority of families were recruited using addresses provided by Medicare Australia (Australia's universal health insurer) and Centrelink (the Australian government income support agency). Other informal means of contact such as word of mouth, local knowledge, and study promotions were used to increase recruitment. Two cohorts were recruited between 2008 and 2009: a kindergarten (K), cohort consisting of 717 children born in 2003, 2004, and 2005 (aged 2.5 to 5 years on recruitment) and a baby (B) cohort consisting of 954 children born in 2006, 2007, and 2008 (aged 6 months to 2 years on recruitment).^33^ The total sample of 1,671 children represented an estimated 5% of Aboriginal and Torres Strait Islander children aged between 6 months and 5 years at the time of recruitment.^34^

Following recruitment and initial data collection in 2008, the children (and families) are followed up with annually. The respondent parent/carer in the survey is the person who best knows the child; most of the time it is the birth mother (93%) and less often the father (2%) or another person such as an aunt or foster carer (5%).^30^

### Ethics

Ethical clearance for the LSIC study was obtained from the Human Research Ethics Committee (HREC) of the Australian Government Department of Health. In addition, for each data collection site, ethics approval was obtained through the relevant state or territory HRECs (in accordance with the 2003 National Health and Medical Research Council and Australian Institute of Aboriginal and Torres Strait Islander Studies guidelines). State and territory departments of education and Catholic dioceses were also consulted to gain permission and support for preschool and schoolteachers to complete questionnaires about the LSIC children involved in the study. The lead researcher of any group seeking to use LSIC data is required to sign a data integrity statement covering 5 core principles that outline the process for respectful use of LSIC data. This was done (written communication, September 2015).

### Data Items

LSIC measures a large range of constructs related to child and family well‐being. Data items representing the risk factor domains described by Fryers and Brugha were identified in 3 main ways: (1) matching where possible the items used in Guy et al,^30^ (2) scanning the LSIC data dictionary for relevant items to match to each domain, and (3) reviewing the LSIC literature to find examples of items used in previous studies and reports.^31^, ^32^, ^33^, ^34^, ^35^, ^36^

### Definition of Risk

Where data items were taken from questionnaires with existing validated scoring protocols and recommended cutoffs, these were used to define risk. For continuous items without established cutoffs, risk threshold was defined by selecting a point on the response scale corresponding to regular or frequent risk exposure. For example, the primary bullying question has a 6‐point response scale: [1] yes always, [2] yes a little bit, [3] sometimes more yes, [4] sometimes more no, [5] no not much, or [6] no never. Those answering 1, 2, or 3 were classified as having been exposed to bullying. For categorical items, the most logical option was usually clear. For example questions about the presence of physical illness had 2 potential responses: “yes” or “no.”

See Table 1 for the mapping of domains onto data items and selection of risk definitions for each data item. In total, 33 risk factors and associated data items were mapped onto the 8 domains from Fryers and Brugha.^10^

**Table 1:** Mapping of Fryers and Brugha (2013)^10^ Risk Domains to LSIC Variables and Risk Definitions Used to Estimate Risk Prevalence

Risk Factor	Ages (Years)	LSIC Variable/Question	Risk Definition
**1. Neurological Vulnerability in Childhood**			
Premature birth	0	Q: How many weeks pregnant (were you)/(was the birth mother) when study child (SC) was born? Or: Do you remember how many weeks early or late you were when SC was born?	birth ≤ 32 weeks
Alcohol use during pregnancy	0	Q: After finding out you were pregnant with SC did you drink any alcohol during the pregnancy?	a “Yes” response
Drug use during pregnancy	0	Q: We aren't after any details here, but after finding out you were pregnant with SC did you use any other substances like smoking marijuana, drinking kava, sniffing petrol, or taking any illicit drugs during the pregnancy?	a “Yes” response
Smoking during pregnancy	0	Q: After finding out you were pregnant with SC did you smoke any cigarettes during the pregnancy?	a “Yes” response
Maternal health problems during pregnancy	0	Q: After finding out you were pregnant with SC, were you told by a doctor or nurse that you or the baby had any problems (eg, diabetes, blood pressure, preeclampsia, low iron, or depression)?	mother experienced ≥ 1 health problems
Developmental delay	3‐10	Measured using LSIC‐developed index incorporating cognitive, behavioral, speech and physical items. Scored on a 2‐point scale: [1] Yes or [0] No.	a “Yes” response
Intelligence	6‐10	Intelligence measured using Matrix Reasoning test, from the Wechsler Intelligence Scale for Children.^55^ Test generates score between 1 and 19. Low scores suggest poor visual concept formation, poor or rigid visual reasoning, or poor concentration.^55^	a score ≤ 7^29^, ^30^
**2. Psychological Disturbance AND Disturbed Behaviors**			
Emotional and behavioral problems	4‐10	Measured using the SDQ total problems score from questionnaire completed by the parent. The SDQ comprises 4 subscales: conduct, hyperactivity, emotional problems, and peer problems. Cutoff scores for total problems and subscales differentiating normal, borderline, and abnormal scores were taken from the literature.^56^	a score in the abnormal range (ie, score ≥ 17)
Conduct problems	4‐10	Measured using the SDQ conduct problems subscale score.	score in the abnormal range (≥ 4)
Hyperactivity	4‐10	Measured using the SDQ hyperactivity subscale score.	a score in the abnormal range (≥ 7)
Emotional problems	4‐10	Measured using the SDQ emotional problems subscale score.	a score in the abnormal range (≥ 5)
Peer problems	4‐10	Measuring using the SDQ peer problems subscale score.	a score in the abnormal range (≥ 4)
**3. Personality Features**			
Low self‐confidence	8‐10	Q: How often do you feel proud of (good about) something you have done? Scored on a 3‐point scale: [1] Lots of time, [2] Sometimes, or [3] Hardly.	a score = 3
**4. School Performance**			
Literacy	8‐10	Teacher‐reported 10‐item scale on child's knowledge, behavior, and skills in language and literacy: contributes relevant information to classroom discussions understands and interprets a story or other text read to him/her reads words with regular vowel sounds reads words with irregular vowel sounds reads age‐appropriate books independently with comprehension reads age‐appropriate books fluently able to write sentences with more than 1 clause composes a story with a clear beginning, middle, and end demonstrates an understanding of some of the conventions of print uses the computer for a variety of purposes Each item is scored on a 5‐point scale: [1] Not yet, [2] Beginning, [3] In progress, [4] Intermediate, or [5] Proficient.	a mean score ≤ 2
Approach to learning	4‐10	Teacher‐reported 6‐item scale on child's approach to learning: keeps belongings organized shows eagerness to learn new things works independently easily adapts to changes in routine persists in completing tasks pays attention well Each item scored on a 4‐point scale: [1] Never, [2] Sometimes, [3] Often, or [4] Very often.	a mean score ≤ 2
Numeracy	8‐10	Teacher‐reported 8‐item scale on child's knowledge, behavior, and skills with numbers: can continue a pattern using 3 items demonstrates an understanding of place value models, reads, writes, and compares whole numbers counts change with 2 different types of coins surveys, collects, and organizes data into simple graphs makes reasonable estimates of quantities measures to the nearest whole number using common instruments uses a variety of strategies to solve math problems Each item scored on a 5‐point scale: [1] Not yet, [2] Beginning, [3] In progress, [4] Intermediate, or [5] Proficient.	a mean score ≤ 2
**5. Childhood Adversity**			
Physical health	0‐10	Physical health measured as a “yes” or “no” to the following health problems: eye, ear, skin, or other health problems such as asthma, chest infections, hay fever, tonsillitis, diarrhea, allergies, rheumatic fever, rheumatic heart disease, anemia, physical growth (underweight, overweight).	≥ 1 health problem
Disability	2‐10	Measured using a set of LSIC‐developed items to ask about intellectual disability, specific learning disability, autism spectrum disorder, physical disability, acquired brain injury, neurological problems, speech, and psychiatric disabilities.	scoring “Yes” for any of the items
Injury	4‐10	Measured using LSIC‐developed index from items that ask if the SC ever had burn or scald, dislocation or internal head injury, sprain or strain, concussion or internal head injury, internal injury excluding head, accidental poisoning, dental injury, near drowning, and dog bite.	scoring “Yes” for any of the items
Stressful life events	0‐10	Measured using the LSIC index derived from the list of *major events* experienced by the family in the last 12 months. Following is a list of 15 possible events: pregnancy, sickness, death, lost job, arrested or jailed or police problem, divorce, humbugged, mugged, robbed, assaulted, worries about money, alcohol or drug problems, child upset by family arguments, child scared by other people, child cared for by someone else for at least 1 week.	family having experienced ≥ 3 major life events in the last year.^34^
Financial stress	2‐10	An 8‐item yes‐or‐no scale on financial stressors experienced in the last 12 months: could not pay bills on time could not pay housing payments on time went without meals unable to heat or cool home pawned/sold something received assistance from welfare organization child could not do school activities unable to send child to preschool/childcare	having ≥ 3 financial stressors
Experience of racism	2‐10	Q: How often does your family experience racism, discrimination, or prejudice? Scored on a 5‐point scale: [1] Every day, [2] Every week, [3] Sometimes, [4] Only occasionally, or [5] Never or hardly ever.	a score ≤ 3
Socioeconomic disadvantage	0‐10	Measured using the SEIFA^33^, ^54^ captured at each wave. The SEIFA score for disadvantage incorporates, at the small‐area level, attributes such as unemployment, education level, and household income. Low scores on this index indicate a high level of disadvantage. The SEIFA score ranges from 1 to 10.	a SEIFA score = 1
Bullied	6‐10	Q: Do the children at (preschool/school) pick on you? Scored on a 3‐point scale: [1] Yes, [2] Sometimes, or [3] No. Alternate Question for 8½‐ to 10‐year‐old children: Q: Do the children at school pick on (or tease) you? Scored on a 6‐point scale: [1] Yes always, [2] Yes a little bit, [3] Sometimes more yes, [4] Sometimes more no, [5] No not much, or [6] No never.	a score ≤ 2 or a score ≤ 4
**6. Child Maltreatment**			
Domestic violence	2‐10	Question to the parents who were living together: Q: How often do P1 and partner have arguments that include physical violence? Alternate question to the parent living elsewhere: Q: How often are there big fights between you and SC's mum or dad? Scored on a 5‐point scale: [1] Never/almost never, [2] Rarely, [3] Sometimes, [4] Often, or [5] Always/almost always.	a score ≥ 3
**7. Parenting, Parent‐Child Relationships, and Parent Psychological Disturbance**			
Parental hostility	4‐10	Indicative of parental hostility consisting of 4 items: How often do you yell or shout when you are telling off SC for doing something wrong? How often would you make SC stay in (his/her) bedroom for misbehaving? If you tell SC (he/she) will get punished if (he/she) doesn't stop doing something, but (he/she) keeps doing it, how often do you end up punishing (him/her)? If you tell SC (he/she) will get punished if (he/she) doesn't stop doing something, but (he/she) keeps doing it, how often do you end up smacking (him/her)? Each item is scored on a 5‐point scale: [1] Always, [2] Often, [3] Sometimes, [4] Rarely, or [5] Never.	a mean score < 3
Parental warmth	4‐10	Parental warmth consisting of 6 items: How often do you hug or hold SC for no particular reason? How often do you enjoy listening to SC? When SC does something really well, how often do you go out of your way to say how pleased you are? How often do you enjoy doing things together with SC? How often do you feel close to SC when (he/she) is happy? How often do you feel close to SC when (he/she) is upset? Each item is scored on a 5‐point scale: [1] Always, [2] Often, [3] Sometimes, [4] Rarely, or [5] Never	Low parental warmth: a mean score ≥ 3
Parental monitoring	4‐10	Parental monitoring consisting of 3 items: When you tell SC to do something, how often do you make sure (he/she) does it? When SC is playing away from home, how often do you know where (he/she) is and who (he/she) is with? How often do you ask SC where (he/she) is going and what (he/she) is doing when (he/she) leaves the house without you? Each item is scored on a 5‐point scale: [1] Always, [2] Often, [3] Sometimes, [4] Rarely, or [5] Never.	Undermonitoring: a mean score ≥ 3
Parental distress	0‐10	Parental distress scale consisting of 7 items: Have you stopped liking everything that used to be fun? Have you felt like everything is hard work (even little jobs are too much) or felt too lazy to do anything? Have you ever felt so worried that your stomach has got upset? Have you ever felt so worried that it was hard to breathe? Do you get angry or wild real quick? Have you felt so sad that nothing could cheer you up; not even your best friends made you feel better? Do you do silly things without thinking that you feel ashamed about the next day? Each item is scored on a 4‐point scale: [1] Never, [2] Little bit, [3] Fair bit, or [4] Lots.	We recoded the answer to have 0‐Never to 3‐Lots. The risk was defined by having a total score ≥ 6.
**8. Disrupted Families**			
Not living with both parents	0‐10	For waves 1 to 4: Q: Does SC have a natural parent living elsewhere? Scored on a 5‐point scale: [1] Yes, [2] No, [3] Can't do, [4] Birth parent deceased, or [5] Birth parent permanently not involved in SC's life. Alternate question asked in waves 5 and 6: Q: Do both of SC's birth parents live in the household? Scored on a 4‐point scale: [1] Yes, [2] No, [3] Deceased, or [4] Permanently not involved in SC's life.	Waves 1 to 4 risk defined by having a score = 2, 4, or 5. In waves 5 to 6, risk defined as 2, 3, or 4.
Regular couple argument	2‐10	Identified from 3 questions asked to parents living together and to separated parents (based on the last 3 months): How often do you disagree with your partner (or SC's mum or dad) about how to bring up SC? How often do you find it hard to talk to your partner (or to SC's mum or dad)? How often are there big fights between you and your partner (or SC's mum or dad)? Each item scored on a 5‐point scale: [1] Never, [2] Rarely, [3] Sometimes, [4] Often, or [5] Always.	a mean score ≥ 3
Parent with problematic drinking	2‐10	Q: In the last year, how often have you had (females: 3 or more; males: 5 or more) alcoholic drinks in 1 session? Scored on an 8‐point scale: [1] Every day, [2] Nearly every day, [3] Couple of times a week, [4] Once a week, [5] Once a fortnight, [6,7,8] Once a month or less.	a score ≤ 3
Household with drug/alcohol problem	0‐10	Q: Have you or a close family member had an alcohol or drug problem (in the last year)?	a “Yes” response

Abbreviations: SC, study child; SDQ, Strength and Difficulties Questionnaire; SEIFA, Socio Economic Index For Areas; LSIC, Longitudinal Study of Indigenous Children; P1, primary carer.

### Data Analyses

Data from the first 6 waves of LSIC were used, spanning ages from 6 months to 10 years. The sample size was 1,671 in the initial 2008 wave and 1,239 in the 2013 wave, reflecting loss to follow‐up over the 6 years. The analysis was conducted in 3 steps: We first created 6 files containing each wave's observation for each of the variables of interest for the risk definition and for demographic variables such as gender, age, and cohort identification. We then added together all 6 files (constructing a long file). We obtained an analytic sample of 8,378 child‐observations. We defined 5 age groups ranging from 0–1 for the group under 2 years (6 months to under 2 years old) to 8–10 for the group 8 to < 10 years old. We used the generalized estimating equations (GEE) method to compute the prevalence of risk factors by age category. Prevalence was estimated both unadjusted and adjusted for clustering (for repeat observations for each child). The GEE was also used to calculate a multiple risk score. The Appendix gives the details on the total child‐observations in each age category at each wave.

IBM SPSS Statistics 22 software and Microsoft Excel were used in all computations and graphic representations.

#### Individual risk factor prevalence

Each risk factor was rescored as a binary variable indicating either the presence (1) or absence (0) of risk. Prevalence was then estimated at each age group by calculating the percentage of children in each age group for whom the risk factor was present. The process was repeated for each risk factor listed in Table 1 and across the 5 age groups. Prevalence was not reported when the number of child‐observations was less than 40 (reflecting an unintended overlap in the age of the B and K cohorts, not missing data). The prevalence reported is as computed—that is, without adjustment—given the absence of published population weights in the LSIC study. Risk of “ever being exposed” was also computed by searching across the 6 waves for each child for any exposure for each of the 28 risk factors. Ever‐exposed will thus always exceed exposure at a single age group.

#### Missing data

Missing values were treated as follows. When there was an alternate source—for example, from the other parent—that data was used. For composite scales generated by combining data from responses to a number of questions, a score was calculated using the available responses; thus a score was entered when at least one input to that scale was observed. After these procedures, there were between 0.02% and 10.4% missing values depending on the risk factor. Missing variable rates were higher for “regular couple argument,” “domestic violence,” and not living with birth parent (7.2%, 7.9%, and 10.4%, respectively) and increased with age category. For these 3 variables, missing values were replaced by the information reported in the previous or subsequent wave when available. Finally, for all other variables where the rate of missing data was below 5%, missing observations were excluded, in effect assuming observations were missing at random. As the more vulnerable families are likely to be overrepresented in missing data, our estimates of risk prevalence are likely to be conservative. With less than 5% missing data, the possible impact on prevalence estimates would be small.^37^

#### Multiple risk index

Studies consistently find that the number of childhood risk factors or “adverse childhood experiences” predict adverse outcomes—including the likelihood of developing a mental disorder in adulthood.^38^, ^39^, ^40^, ^41^, ^42^, ^43^, ^44^ This association is well described in the Adverse Childhood Experiences study.^3^, ^4^, ^5^, ^6^ Multiple risk indices help discriminate between higher‐ and lower‐risk individuals and are typically created by summing the number of dichotomized risk factors.^38^, ^42^, ^43^

For this study, the multiple risk index was constructed by counting the number of risk factors to which a child was exposed at each age group (see Table 1), but excluding the Strength and Difficulties Questionnaire (SDQ) total problem score (given inclusion of the 4 SDQ subscales). The maximum number of risk factors varied across the ages (11 in infants 6‐months to < 2 years, 18 in 2‐ to 3‐year‐olds, 27 in 4‐ to 5‐year‐olds, 29 for 6‐ to 7‐year‐olds, and 32 in 8‐ to 10‐year‐olds). The multiple risk index calculated for each study child at each wave also included the acquired risk score in utero. The distribution of multiple risk was then computed by age category. We also computed the prevalence of multiple risk factors for those children with an abnormal SDQ total problem score at each age.

## Results

The prevalence of individual risk factors is presented in Table 2, where we report the prevalence of risk at each age range and percentage of children ever exposed (over 6 waves). Results reported are unadjusted since almost no differences were observed in the adjusted and unadjusted estimates.

**Table 2:** Prevalence^a^ of Individual Risk Factors for Adult Mental Illness Across Age Category

Neurological Vulnerability (%)	
Premature birth	3.3
Prenatal risk exposure:	
Alcohol use during pregnancy	22.1
Drug use during pregnancy	7.3
Smoking during pregnancy	49.2
Maternal health problems during pregnancy	31.4

	Age Category					
	0‐1 y (%)	2‐3 y (%)	4‐5 y (%)	6‐7 y (%)	8‐10 y (%)	Ever (%)
Developmental delay		4.3	2.1	2.4	1.7	6.1
Intelligence (matrix reasoning score)				38.8	44.0	53.9
Psychological Disturbance and Disturbed Behavior						
SDQ total problems—abnormal			23.8	22.7	22.7	31.3
SDQ conduct problems—abnormal			41.1	27.3	26.4	46.7
SDQ hyperactivity—abnormal			21.6	25.4	24.0	33.2
SDQ emotional problems			13.3	18.7	18.5	21.8
SDQ peer problems			21.9	25.2	28.2	34.1
Personality Features						
Low self‐confidence					4.6	4.7^b^
School Performance						
Low literacy					20.4	20.4
Low approach to learning			18.2	18.1	18.6	24.7
Low numeracy					14.8	15.2^b^
Child Maltreatment						
Domestic violence		12.7	23.8	16.3	19.7	41.2
Childhood Adversity						
Poor physical health	45.5	55.9	53.0	56.0	53.4	87.8
Physical disability		1.9	2.1	2.5	3.3	6.5
Injury			6.1	4.7	5.3	12.7
Stressful life events (3+)	67.2	70.3	66.1	68.9	68.2	95.0
Financial stress (3+)		26.2	32.2	27.5	29.9	42.8
Family experience of racism		20.7	21.7	18.0	23.0	29.3
Socioeconomic disadvantage	42.7	45.4	44.6	45.4	44.8	56.6
Bullied				40.5	36.4	68.6
Parenting, Parent‐Child Relationship, and Parent Psychological Disturbance						
Parental hostility			32.4	29.8	29.3	56.2
Low parental warmth			0.8	0.9	1.3	1.7
Parental monitoring			4.4	1.3	2.2	5.3
Parental distress	32.4	24.6	28.1	23.0	22.5	52.5
Disrupted Families						
Not living with both parents	42.5	47.9	52.1	57.7	53.3	61.4
Regular couple argument		22.8	26.6	23.5	26.0	31.9
Parent with problematic drinking		24.9	29.5	28.5	35.3	34.9
Household with drug/alcohol problem	22.2	21.5	22.6	24.6	25.0	52.2

Abbreviation: SDQ, Strength and Difficulties Questionnaire.

^a^Adjusted for repeated measures, unadjusted for clustering by region.

^b^Ever is slightly different from the percentage at age 8–10 since it also includes responses from a small number of children (n < 40) in the 6–7 age group for whom the mean prevalence was higher.

Close to 50% of children were exposed to smoking by their mothers during pregnancy, 22% to alcohol intake, and 31% had mothers who experienced at least 1 health problem during pregnancy. About 23% to 24% of children had an SDQ total problems score in the clinical range, varying little between ages 4 and 10. Rates of conduct problems were higher in younger children (41% of 4‐ to 5‐year‐olds) and lower in older children (26% of 6‐ to 10‐year‐olds). Being bullied was reported by 36% of 8‐ to 10‐year‐old children and 41% of 6‐ to 7‐year‐olds, with 69% reporting having been bullied at some time between ages 6 and 10. Relatively few children were identified as having low self‐esteem.

Many indigenous children are exposed to elements of a disrupted family environment. As babies, 43% of children did not live with both birth parents; this increased to 48% by age 2‐3 years. More than 23% were exposed to a high level of regular couple arguments each year, with 32% ever exposed. At ages 2 to 3 years, 13% of children were currently exposed to domestic violence while 24% of 4‐ to 5‐year‐olds were exposed, with 41% ever exposed to domestic violence in the 8 years of data collection. Between 25% and 35% of the children were currently exposed to problematic parental drinking, with 35% of children ever exposed.

The majority of children (> 67%) were exposed to 3 or more major life events in their family (eg, incarceration, death of family member, lost job) in any one year, with 95% ever exposed; and between 26% and 32% of children were living in families reporting at least 3 indicators of financial stress over the previous 12 months, with 43% ever exposed.

For parenting and parent‐child relationships, from 4 years of age, around 30% of children were consistently exposed to parenting that might be viewed as hostile (56% ever exposed), and 22% to 26% lived with parents experiencing a high level of distress (53% ever exposed). Racism (defined by family experiencing racism, discrimination, or prejudice from “sometimes” to “every day”) was experienced by 1 in 5 children in any year, and by 29% of children over the 8 years of data collection.

### Multiple Risk Index

**Figure 1:**
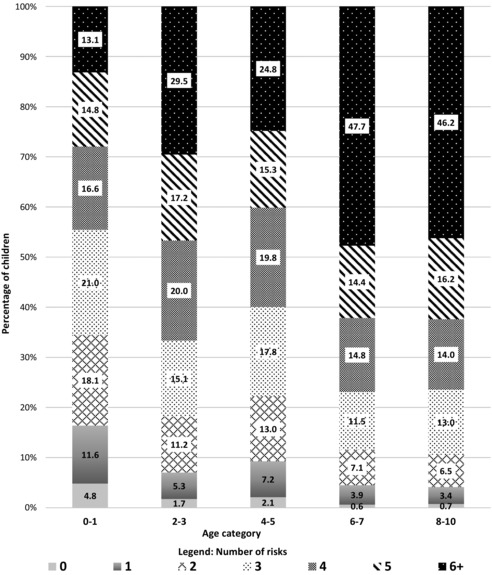
Compounding of Childhood Adversities in Indigenous Children in LSIC (Percentage of Children by Number of Risks)

Among children aged 8‐10 years, 46% had 6 or more defined risk factors of mental illness while only a very small proportion (from < 1% to 5%) were identified as risk free (Table 3 and Figure 1). The prevalence of 6 or more risks increased from 30% in children aged 2‐3 years of age to 46% by ages 8‐10 years (see Figure 1).

**Table 3:** Prevalence of Multiple Risk by Age Group

	Age Category				
Number of Risk Factors	6 mo to < 2 y (%)	2 y to < 4 y (%)	4 y to < 6 y (%)	6 y to < 8 y (%)	8 y to 10 y (%)
0	4.8	1.7	2.2	0.6	0.8
1	11.6	5.3	7.2	3. 9	3.4
2	18.1	11.2	13.0	7.1	6.5
3	21.0	15.1	17. 8	11.5	13.0
4	16.6	20.0	19.8	14.8	14.0
5	14.8	17.2	15.3	14.4	16.2
6	8.7	12.4	10.6	12.2	13.8
7	2.8	7.6	7.0	11.5	10.2
8	1.2	4.2	4.3	8.7	8.8
9	0.5	3.0	1.6	5.8	5.2
≥ 10	0.00	2.4	1.4	9.6	8.1
Sum	100	100	100	100	100
Maximum n of Risks	11	18	27	29	32
Total Child‐Observation Time‐Points	830	1,784	2,739	1,956	1,069

**Figure 2:**
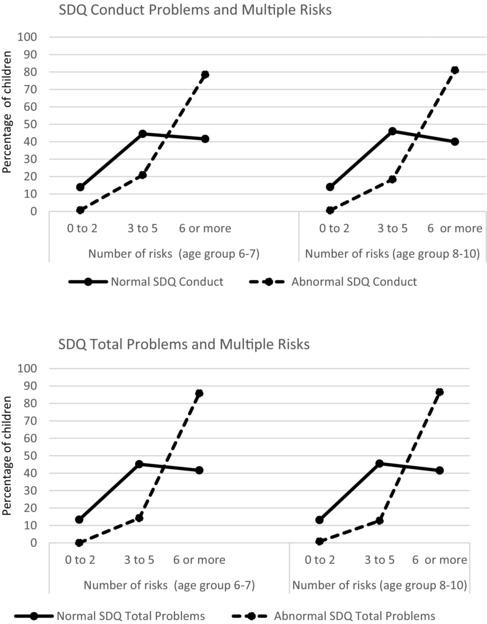
Children with SDQ Conduct and Total Problems scores in the Normal or Abnormal Range and Mean Number of Risks at Ages 6 to 7 Years and 8 to 10 Years

For children aged 8‐10 years with an SDQ total problem score in the clinical range, indicating likely psychological distress, the vast majority (87%) also had 6 or more risk factors. Similarly for children with clinical levels of conduct problems or hyperactivity, 82% had 6 or more risks for adult mental illness (Figure 2). A similar result was observed for children aged 6‐7 (see Figure 2). For children with a normal score on the SDQ total problems and SDQ conduct scales the proportion of those with 6 or more risk factors is 40% (in both the 6‐7 and 8‐10 age groups). The difference is statically significant (*P* < 0.0001).

## Discussion

This study builds on the small body of research on adversities in Indigenous Australian children, but with a specific focus on the risks for mental illness. As far as we are aware, it is the first study to estimate exposure to multiple risks from infancy through age 10 in Indigenous Australian children, and possibly the first study for First Nations children internationally. The findings have important implications for how we think about support services for Indigenous children and families.

We found that by age 8, only 4% of Indigenous children in LSIC had ≤ 1 adversity and that 46% of children aged 8 to 10 years had 6 or more risk factors for mental illness. The life trajectory of a child exposed to none or just 1 risk factor is likely very different to that of a child exposed to 6 or more.

Indigenous children in LSIC are exposed to considerably higher rates of adversity than a general Australian sample from the Longitudinal Study of Australian Children (LSAC).^30^ For example, more than 67% of Indigenous children are in families experiencing 3 or more major stressful life events in the previous 12 months, compared to just 14% of children in LSAC (using the same definitions). Exposures in utero are also higher: alcohol use during pregnancy (2 times the LSAC sample), smoking during pregnancy (3.75 times), and maternal mental ill health in pregnancy (2 times). Taking the 6‐ to 7‐year‐old cohort, exposure to domestic violence reported in LSIC was 11 times that for the LSAC sample, poor child physical health (5.3 times), having 3 or more indicators of family financial stress (3.5 times), parental distress (1.5 times), and household with drug/alcohol problem (9.8 times). Experience of child psychological distress was also high. For each of these variables, definitions used in both surveys were the same or nearly so.

With the far higher rate of multiple adversity, we find 22.5% of indigenous children between the ages of 4 and 10 years report high levels of psychological distress (using the SDQ total problems score), compared with just over 8% of children of the same age in LSAC.^29^ Other studies put rates of psychological distress in Australian children higher, across a broad age range.^21^, ^44^, ^45^, ^46^

This study finds that many Indigenous Australian children, from infancy, are at significant risk of developing mental illness based on the high prevalence of accumulated childhood stressors. We also found that children experiencing current psychological distress (identified by SDQ scores in the clinical range) had almost always been exposed to very high rates of childhood adversities—consistent with the developmental conceptualization of mental illness that underpins this study.

In spite of the high levels of adversity, LSIC children generally report a positive sense of self, suggestive of resilience. We also find that low parental warmth is rarely identified as an issue, a favorable parenting attribute.

### Limitations

While risk factors were identified from a very large global review,^10^ the included studies were predominantly (although not exclusively) of nonindigenous populations. We thus explored whether these risk factors are likely relevant for indigenous families. Indigenous families often have different family structures (eg, involvement of extended family) that may suggest a factor such as “living with both birth parents” does not constitute a risk. However, Australian research^22^ has found that Indigenous children most at risk of clinically significant emotional or behavioral difficulties do have a very similar risk profile to nonindigenous children, such as exposure to poor parenting, poor family functioning, and sole parent care, which aligns well with the domains we used in this study. We also note that the domains identified in Fryers and Brugha are consistent with what would be predicted from a developmental origins understanding of mental illness; that is, they have a sound theoretical basis.^47^

In terms of the generalizability of results across Australian Indigenous populations, we note that the sampling method was designed to achieve a mix of community settings, reflective of the broad Australian experience, and that the use of Medicare provides the most complete database. Where comparable data are available, in general findings are consistent. For example, from LSIC we observe 67%‐70% of children across all age categories are in families experiencing 3 or more stressful life events in a 12‐month period, identical to the findings of the WAACHS, which reported 70% of indigenous families experienced 3 or more major stressful life events in the previous 12 months.^22^ Our study also found that 22%‐26% of children were exposed to parental high distress. This estimate is comparable to (although somewhat lower than) the prevalence of psychological distress in Aboriginal and Torres Strait Islander people aged 16 years and overreported in other Australian studies at 31%.^5^, ^48^

The identification and measurement of risk was limited to the set of variables available in LSIC. The mapping of LSIC variables to the risk factor domains identified in Fryers and Brugha^10^ was not perfect. For example, there were no direct questions on child abuse and neglect, although there was a specific question on domestic violence, the witnessing of which is a designated form of abuse.^38^, ^49^, ^50^ Other questions on parenting quality were informative although not standard. Our definitions of risk threshold were informed by published cut‐points or, where not available, drew on the inherent meaning in the response options. This necessarily introduces a level of subjectivity into the analysis.

Although we took steps to reduce the impact of missing data, the prevalence estimates are subject to error. An underestimate of the real extent of risk in the population of Indigenous children is the probable direction of bias, through a combination of bias in missing data (the more vulnerable and distressed families are likely to have more missing data); and the possibility of self‐report bias (the potential for under‐reporting of undesirable behaviors).^37^, ^51^

### Implications and Interpretations

The clustering and accumulation of modifiable risk factors for poor mental health, starting in utero, highlights the need for a multisectoral approach capable of modifying a complex set of risk factors and identifying and addressing emerging child psychological distress, starting early in life. This means an integrated service model incorporating maternal and perinatal health services that continues through childhood across child and family mental health, comprehensive primary care, early childhood, family support services, and the broader human service canvas (eg, income support, housing, child protection, education). Critically, these services need to work together in a way that acknowledges complexity—that distressed children and families commonly present with multiple risk factors, such as disrupted family relationships, socioeconomic stress, other health problems, and histories of profound trauma.

Building a family support service system that can genuinely cater for complexity is a requirement for all vulnerable families, but nowhere is this most urgent than for Indigenous Australian families, whose greater exposure to adversity is not limited to the social determinants reported here, but includes ongoing and historical traumas related to colonization, separation from families, removal from country, intergenerational trauma, language and culture loss, loss of connection with land, systemic racism, disempowerment, marginalization, and discrimination. A recent paper from the Study of Environment on Aboriginal Resilience and Child Health finds psychological distress to be common in carers of Aboriginal children in Australia and calls for increased funding to Aboriginal Community Controlled Health Services for delivery of mental health services and a focus on social connectedness.^52^

While the data presented in this paper is challenging, we note a number of positive developments with regards to Aboriginal and Torres Strait Islander health and research frameworks and policy commitments. The most recent National Aboriginal and Torres Strait Islander Social Survey (ABS, 2014‐2015), the fourth of its type, noted significant improvement across a range of indicators of education, health, and housing since the last survey.^53^ In terms of action, the publication in 2014 of the second edition of *Working Together: Aboriginal and Torres Strait Islander Mental Health and Wellbeing Principles and Practice* 54 synthesized a large body of work on Indigenous mental health for researchers and clinicians including models for working with families and communities to heal the trauma of past wrongs. Core discussion elements—such as need for a much broader and holistic definition of mental health that encompasses connection to family, community, land, culture, and spirit; a trauma‐informed approach to healing families and communities; and a focus on engaging communities to develop and implement their own service models—are pertinent to thinking about addressing complexity in all vulnerable families. In this regard, nonindigenous researchers and service systems have much to learn from this emerging literature on Indigenous mental health.

## Conclusion

Our results constitute important evidence on the high levels of current psychological distress combined with multiple risk exposure in Indigenous Australian children. This evidence should guide the development of preventive strategies to better support these highly vulnerable families and children, from as early as possible in the life course. An effective integrated service system that can address complexity and multiple adversity from conception through to adolescence is urgently needed to address the high rates of current psychological distress in Indigenous Australian children and offer the prospect of reducing the future burden of mental illness. Exactly what that service response should look like will need to be developed in conjunction with indigenous communities, but a capacity to address the high load of adversities in children and parents using a trauma‐informed and compassionate lens, while offering practical support at the same time, will be central.

## Number of Observations From Each Wave by Age Category

**Table:**

	Age Category, y							
	0‐1	2‐3	4‐5	6‐7	8‐10	All	Lost to Follow‐Up	Total
Wave 1	790	233	647	1	0	1,671	0	1,671
Wave 2	40	836	544	103	0	1,523	148	1,671
Wave 3	0	680	191	532	1	1,404	267	1,671
Wave 4	0	35	717	453	78	1,283	388	1,671
Wave 5	0	0	605	164	489	1,258	413	1,671
Wave 6	0	0	35	703	501	1,239	432	1,671
**Total number of person‐observations**	**830**	**1,784**	**2,739**	**1,956**	**1,069**	**8,738**	**1,648**	**10,026**
